# Detection of AML-specific *TP53* mutations in bone marrow–derived mesenchymal stromal cells cultured under hypoxia conditions

**DOI:** 10.1007/s00277-019-03680-4

**Published:** 2019-04-02

**Authors:** Marian Müller, Ricarda Graf, Karl Kashofer, Susanne Macher, Albert Wölfler, Armin Zebisch, Andelko Hrzenjak, Ellen Heitzer, Heinz Sill

**Affiliations:** 1 Division of Hematology, Medical University of Graz, Auenbruggerplatz 38, A-8036 Graz, Austria; 2 Institute of Human Genetics, Diagnostic and Research Center for Molecular Biomedicine, Medical University of Graz, Graz, Austria; 3 Institute of Pathology, Medical University of Graz, Graz, Austria; 4 Department for Blood Group Serology and Transfusion Medicine, Medical University of Graz, Graz, Austria; 5 Division of Pulmonology, Medical University of Graz, Graz, Austria; 6 Ludwig Boltzmann Institute for Lung Vascular Research, Graz, Austria

Dear Editor,

*TP53* mutations are early events in the pathogenesis of acute myeloid leukemia (AML) and *TP53*-mutated AML has recently been classified as a distinct subentity [1–3]. An increasing number of reports postulate that the bone marrow (BM) microenvironment of patients with myeloid malignancies contributes to both leukemogenesis and therapeutic resistance [4]. As disease-specific, somatic aberrations have been reported in cells of the BM microenvironment in these disorders [5, 6], we hypothesized that BM-derived mesenchymal stromal cells (BM-MSCs) are also affected by leukemia-specific mutations in patients with *TP53*-mutated AML.

The study was approved by the ethics committee of the Medical University of Graz, Austria, and written informed consent was obtained from all patients. Diagnostic, vitally frozen BM specimens from 13 AML patients with somatic *TP53* mutations were used for BM-MSC culture (Supplementary Table 1) [7]. One specimen from a patient with Li-Fraumeni-syndrome suffering from therapy-related AML served as a positive control. In accordance with previous reports, these leukemia specimens revealed a complex karyotype (12/14; 86%) and a paucity of cooperating gene mutations (median, 1; range, 0–3) [3]. As outlined in detail in the “Supplementary Methods,” ex vivo culture of mononuclear BM cells was performed under low oxygen conditions (3% pO_2_ and 5% CO_2_ at 37 °C) with the addition of human platelet lysate. Adherent cells representing BM-MSCs were cultivated up to a maximum of 4 passages. To obtain pure cell populations, they were further subjected to cell sorting by FACS (FACSAria, BD) using the human monoclonal antibodies CD 73, CD105 (Bioscience), CD90 (Biolegend), and CD34 (Biolegend), CD45, CD14, and HLA-DR (all Beckman Coulter), respectively. In addition, their adipogenic, chondrogenic, and osteogenic differentiation capacity as a characteristic feature of BM-MSCs was demonstrated (Supplementary Fig. 1) [8]. Patient-specific *TP53* and cooperating mutations were analyzed in both AML and purified BM-MCS specimens, using the error corrected, high-resolution “Safe-Sequencing System” method as described previously [1, 3]. In AML specimens, somatic *TP53* and cooperating mutations were found at variant allele frequencies (VAFs) between 1.5 and 91.2%. In purified BM-MSCs, the leukemia-specific *TP53* mutation was detected in 2/13 patients (15%) at VAFs of 0.2% each and confirmed using biological replicates (0.2% and 0.1%, respectively) (Fig. 1). However, apart from one single nucleotide polymorphism in *TET2 (*c.100C > T, p.L34F [rs111948941], sample #7479), no leukemia-specific, cooperating mutation was detected in BM-MSCs in any of the specimens analyzed (Supplementary Table 2).

**Fig. 1:**
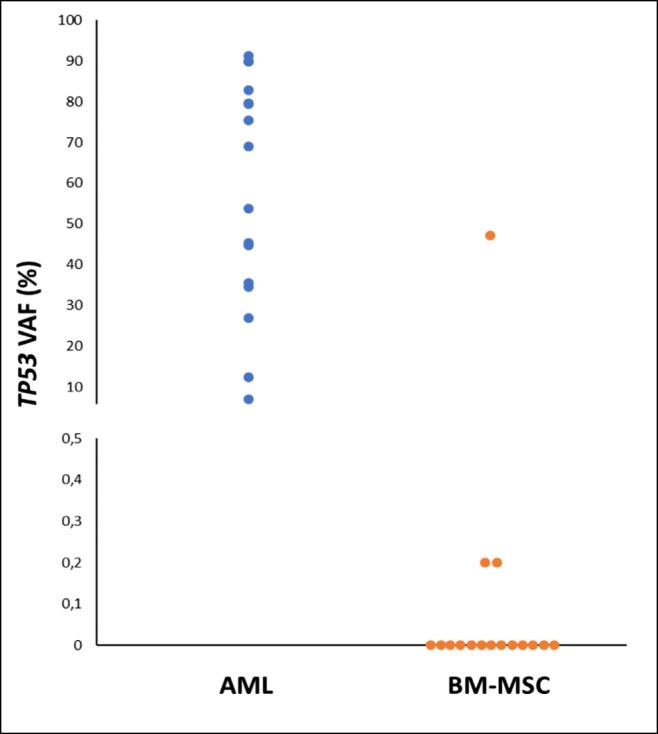
Variant allele frequencies (VAFs) from primary leukemia specimens and purified bone marrow–derived mesenchymal stromal cells (BM-MSCs) from patients with *TP53*-mutated acute myeloid leukemia (AML). The BM-MSC specimen with a VAF of 47.1% was derived from a patient with Li-Fraumeni syndrome suffering from therapy-related AML serving as a positive control

The detection of somatic, leukemia–specific *TP53* mutations in BM-MSCs of AML patients may indicate that these mutations have arisen in common mesodermal ancestors of hematopoietic stem and progenitor cells and BM-MSCs [9]. It further supports the concept of *TP53* mutations being early events of acute myeloid leukemogenesis. The demonstration of BM-MSCs affected by leukemia-specific mutations—albeit at low VAFs—might also have practical implications as these cell types are increasingly used as a source of germline, control DNA [10]. Future work will focus on the functional role of the bone marrow microenvironment in this distinct AML subentity.

## Electronic supplementary material

ESM 1 (DOCX 331 kb)

## References

[1] Lal R, Lind K, Heitzer E, Ulz P, Aubell K, Kashofer K, Middeke JM, Thiede C, Schulz E, Rosenberger A, Hofer S, Feilhauer B, Rinner B, Svendova V, Schimek MG, Rucker FG, Hoefler G, Dohner K, Zebisch A, Wolfler A, Sill H (2017). Somatic TP53 mutations characterize preleukemic stem cells in acute myeloid leukemia. Blood..

[2] Papaemmanuil E, Gerstung M, Bullinger L, Gaidzik VI, Paschka P, Roberts ND, Potter NE, Heuser M, Thol F, Bolli N, Gundem G, Van Loo P, Martincorena I, Ganly P, Mudie L, McLaren S, O’Meara S, Raine K, Jones DR, Teague JW, Butler AP, Greaves MF, Ganser A, Dohner K, Schlenk RF, Dohner H, Campbell PJ (2016). Genomic classification and prognosis in acute myeloid leukemia. N Engl J Med.

[3] Prochazka KT, Pregartner G, Rucker FG, Heitzer E, Pabst G, Wolfler A, Zebisch A, Berghold A, Dohner K, Sill H (2019). Clinical implications of subclonal TP53 mutations in acute myeloid leukemia. Haematologica..

[4] Morrison SJ, Scadden DT (2014). The bone marrow niche for haematopoietic stem cells. Nature..

[5] Garcia-Montero AC, Jara-Acevedo M, Alvarez-Twose I, Teodosio C, Sanchez-Munoz L, Muniz C, Munoz-Gonzalez JI, Mayado A, Matito A, Caldas C, Morgado JM, Escribano L, Orfao A (2016). KIT D816V-mutated bone marrow mesenchymal stem cells in indolent systemic mastocytosis are associated with disease progression. Blood..

[6] Azuma K, Umezu T, Imanishi S, Asano M, Yoshizawa S, Katagiri S, Ohyashiki K, Ohyashiki JH (2017). Genetic variations of bone marrow mesenchymal stromal cells derived from acute leukemia and myelodysplastic syndrome by targeted deep sequencing. Leuk Res.

[7] Olipitz W, Hopfinger G, Aguiar RC, Gunsilius E, Girschikofsky M, Bodner C, Hiden K, Linkesch W, Hoefler G, Sill H (2002). Defective DNA-mismatch repair: a potential mediator of leukemogenic susceptibility in therapy-related myelodysplasia and leukemia. Genes Chromosom Cancer.

[8] Dominici M, Le Blanc K, Mueller I, Slaper-Cortenbach I, Marini F, Krause D, Deans R, Keating A, Prockop D, Horwitz E (2006). Minimal criteria for defining multipotent mesenchymal stromal cells. The International Society for Cellular Therapy position statement. Cytotherapy..

[9] Ratajczak MZ (2015). A novel view of the adult bone marrow stem cell hierarchy and stem cell trafficking. Leukemia..

[10] Mujahed H, Jansson M, Bengtzen S, Lehamnn S (2017). Bone marrow stroma cells derived from mononuclear cells at diagnosis as a source of germline control DNA for determination of somatic mutations in acute myeloid leukemia. Blood Cancer J.

