# Supplementary material for: Detection of AML-specific TP53 mutations in bone marrow–derived mesenchymal stromal cells cultured under hypoxia conditions
Source: Ann Hematol. 2019 Apr 2;98(8):2019–20. doi: 10.1007/s00277-019-03680-4 (PMC6647597; doi:10.1007/s00277-019-03680-4)
Supplement: Supplementary file 1 — (DOCX 331 kb) [file 277_2019_3680_MOESM1_ESM.docx]

## Supplementary Data

**Detection of AML-specific *TP53* mutations in bone marrow-derived mesenchymal stromal cells cultured under hypoxia conditions**

Marian Müller,^1^ Ricarda Graf,^2^ Karl Kashofer,^3^ Susanne Macher,^4^ Albert Wölfler,^1^ Armin Zebisch,^1^ Andelko Hrzenjak,^5,6^ Ellen Heitzer^2^ and Heinz Sill^1^

^1^Division of Hematology, Medical University of Graz, Graz, Austria

^2^Institute of Human Genetics, Diagnostic and Research Center for Molecular Biomedicine, Medical University of Graz, Graz, Austria

^3^Institute of Pathology, Medical University of Graz, Graz, Austria

^4^Department for Blood Group Serology and Transfusion Medicine, Medical University of Graz, Graz, Austria

^5^Division of Pulmonology, Medical University of Graz, Graz, Austria

^6^Ludwig Boltzmann Institute for Lung Vascular Research, Graz, Austria

**Patients and Methods**

*Primary leukemia specimens*

At the Division of Hematology, Medical University of Graz, Graz, Austria (MUG), diagnostic bone marrow (BM) specimens from patients with acute myeloid leukemia (AML) are processed by Ficoll-Hypaque density gradient centrifugation and vitally frozen in liquid nitrogen. They are molecularly characterized by targeted deep sequencing assessing the coding regions of the *CEBPA*, *BCOR*, *DDX41*, *DNMT3A*, *ETV6*, *GATA2*, *NF1*, *PHF6*, *SF3B2*, *SFRP1*, *SRP72*, *STAG2*, *TP53* and *ZRSR2* genes, respectively, as well as mutational hot-spots of *NPM1, ASXL1, BRAF, CALR, CBL, CSF3R, ETNK1, EZH2, FLT3, IDH1, IDH2, JAK2, KIT, KRAS, MPL, NRAS, PTPN11, RUNX1, SETBP1, SF3B1, SRSF2, STAT3, TET2, U2AF1* and *WT1* using the Ion Torrent Ampliseq Panel for “Myeloid Neoplasms”.

*Isolation of bone marrow-derived mesenchymal stromal cells (BM-MSC)*

A total of 14 BM specimens from patients with newly diagnosed *TP53* mutated AML were used for this study (Supplementary Table 1). Between 1.0x10^6^ and 9.5x10^6^ cryopreserved, mononuclear cells (MNCs) were thawed per specimen, washed with calcium-free phosphate buffered saline (PBS) and re-suspended in αMEM (Sigma) supplemented with 10% human platelet lysate (provided by Department for Blood Group Serology and Transfusion Medicine, MUG), 1% L-Glutamine solution (Sigma), 1% Penicillin-Streptomycin (Sigma) and 2 U/ml Heparin (Biochrom) in accordance with previously published protocols.^1^

For *ex vivo* expansion, BM-MNCs were seeded in tissue flasks at a density of 0.8 x10^5^ to 1.6x10^5^ cells/cm^2^ and cultured under low oxygen conditions (3% pO_2_ and 5% CO_2_ at 37°C). After 72 hours, cell culture medium and non-adherent cells were removed. Adherent cells were washed once with calcium-free PBS. Fresh culture medium was added to adherent cells which were cultivated for further 3 to 5 days (1^st^ passage). Fifty percent of culture medium was exchanged every other day. When cells reached 70%-90% confluence, they were detached using TrypLE Express (Gibco) and transferred to a new flask. Adherent cells were cultivated up to maximum of 4 passages.

*Cell sorting*

Cultured BM-MSCs were further sorted by FACS (FACSAria, BD) in cooperation with the core facility “Imaging/Flow Cytometry” at the Center for Medical Research, MUG. Re-suspended cells were labelled by using a broad spectrum of monoclonal antibodies (MoAbs) as previously described:^2^ CD 73, CD105 (Bioscience), and CD90 (Biolegend) were used as positive markers and CD34 (Biolegend), CD45, CD14 and HLA-DR (all Beckman Coulter) as negative markers, respectively. Detached cells were blocked with blocking buffer (10% FBS in calcium-free PBS) for 10 minutes on ice, subsequently re-suspended in staining buffer (3% FBS in calcium-free PBS) with a maximum cell concentration of 1.0x10^7^ cells/ml and incubated with MoAbs for 25 min at 4°C in the dark.

*Adipogenic, chondrogenic and osteogenic differentiation*

The adipogenic, chondrogenic and osteogenic differentiation capacity of BM-MSCs were tested using commercially available assays (MesenCult™, STEMCELL Technologies) (Supplementary Figure 1). For all three assays, *in-vitro* cultures were performed at 20% pO_2_.

For adipogenic differentiation, BM-MSCs were plated at a density of 6.0x10^3^ cells/cm^2^ and cultured in the MSC medium described above until they reached confluence of 90%-100%. Thereafter, the medium was replaced by “MesenCult™ Adipogenic Differentiation Medium” (#05412 Human) and cells were cultured for additional 20 days with a medium change every 3 days. During that time, lipid vacuoles were formed. Adipogenic differentiation was assessed by Oil Red O staining.

For chondrogenic differentiation, 2.0x10^6^ BM-MSCs were re-suspended in 2 ml of “MesenCult™-ASF Chondrogenic Differentiation Medium” (#05455). Cell suspensions of 0.5 ml were transferred into Falcon tubes and centrifuged for 5-10 min at 300x g and BM-MSC incubated at 5% CO_2_ and 37°C for 3 days. Incubation was carried out for 6 to 21 days with the medium changed every 3 days. When cells have reached chondrogenic differentiation, pellets were fixed in 10% formalin for 30 minutes at room temperature, followed by paraffin embedding. Sections of 6 μm were stained with Alcian Blue and Nuclear Fast Red.

For osteogenic differentiation, BM-MSCs were plated in triplicates in six-well plates at a cell density of 6.0x10^3^ cells/cm^2^. When reaching confluence of 70%-80%, the culture medium was replaced by “MesenCult™ Osteogenic Stimulatory Medium” (#05465 without β-glycerophosphates). During the subsequent cultivation period of 1 week, BM-MSCs formed a multilayer and β-glycerophosphates were added. The cells were then cultivated for further 4 weeks with a medium change every 3 days. During that time, calcium deposition could be observed. Osteogenic differentiation was finally assessed by Alizarin Red S staining.

*Error-corrected, next generation sequencing*

Patient-specific *TP53* and cooperating mutations were analyzed in purified BM-MSCs and re-analyzed in leukemia specimens, respectively, using the high-resolution Safe-Sequencing System (Safe-SeqS) method as previously described.^3-5^ Primers spanning the respective mutations were designed using primer 3 software (http://bioinfo.ut.ee/primer3-0.4.0/). 10-20 ng of DNA were amplified using 1U Phusion DNA Polymerase (Thermo Fisher), 0.25mM dNTPs and 02.µM amplicon-specific primers in 10 cycles of amplicon-specific PCR. To remove first-round primers, the PCR products were purified using Ampure XP beads (Beckman Coulter) and eluted in 15 µl nuclease-free H_2_O. In a second round of PCR, Illumina specific adapters and indices were attached to the 5’ ends for 35 cycles. After the second round of amplification, PCR fragments were again purified using Ampure XP beads (Beckman Coulter) and eluted in 12 µl of nuclease-free H_2_O. For quality control and quantification, samples were run on an Agilent Bioanalyzer DNA 7500 chip (Agilent Technologies). All samples from one patient were pooled equimolarly and sequenced on an Illumina MiSeq in a 2x150 bp paired-end run.

Generated reads were grouped to read families according to the unique identifier (UID) added to the target-specific primer. Reads containing an "N" in the UID were discarded. After grouping, a consensus sequence of each read family was generated by picking a base that occurs in at least 80% of all the reads assigned to a family at that position. If no consensus was found, "N" was used as the consensus output base at that position. Based on the grouping output, a new fastq-file was generated form the consensus sequences of each read family comprising at least 5 reads and containing a maximum of 2 N positions at the forward and reverse consensus sequence, respectively. If after read collapsing, the sequencing depth was less than 1000x and no mutation was detected, we additionally included read families with 1-4 reads for assessing the presence of a respective mutation. Forward and reverse sequences were merged using FLASH and the resulting FastQ file was aligned to the human reference genome (hg19) using BWA and samtools. Alignments were visualized in IGV to detect variations.

To test the limit of detection of Safe-SeqS, we used a serial dilution of a cell line harboring the *TP53* mutation c.743G>A, p.R248Q. This *TP53* mutation could be detected at a variant allele frequency as low as 0.2%. Moreover, sequencing of genomic DNA of 10 *TP53* wild-type specimens revealed no false-positive reads when considering only read families comprising of at least 5 reads and an error rate of 0.88% when all read counts were taken into account, respectively.

In cases of detection of leukemia-specific mutations in BM-MSCs, biological replicates were analyzed using DNA extracted from a different passage of BM-MSCs.

*References*

1. Schallmoser K, Bartmann C, Rohde E, Reinisch A, Kashofer K, Stadelmeyer E, Drexler C, Lanzer G, Linkesch W, Strunk D. Human platelet lysate can replace fetal bovine serum for clinical-scale expansion of functional mesenchymal stromal cells. Transfusion. 2007;47(8):1436-1446.

2. Dominici M, Le Blanc K, Mueller I, Slaper-Cortenbach I, Marini F, Krause D, Deans R, Keating A, Prockop D, Horwitz E. Minimal criteria for defining multipotent mesenchymal stromal cells. The International Society for Cellular Therapy position statement. Cytotherapy. 2006;8(4):315-317.

3. Kinde I, Wu J, Papadopoulos N, Kinzler KW, Vogelstein B. Detection and quantification of rare mutations with massively parallel sequencing. Proc Natl Acad Sci U S A. 2011;108(23):9530-9535.

4. Lal R, Lind K, Heitzer E, Ulz P, Aubell K, Kashofer K, Middeke JM, Thiede C, Schulz E, Rosenberger A, Hofer S, Feilhauer B, Rinner B, Svendova V, Schimek MG, Rucker FG, Hoefler G, Dohner K, Zebisch A, Wolfler A, Sill H. Somatic TP53 mutations characterize preleukemic stem cells in acute myeloid leukemia. Blood. 2017;129(18):2587-2591.

5. Prochazka KT, Pregartner G, Rucker FG, Heitzer E, Pabst G, Wolfler A, Zebisch A, Berghold A, Dohner K, Sill H. Clinical implications of subclonal TP53 mutations in acute myeloid leukemia. Haematologica. 2019;104(3):516-523.

**Results**

| **Sample #** | **Sex** | **Age** | **Type of leukemia** | **WBC [G/l]** | **Cytogenetics** |
| --- | --- | --- | --- | --- | --- |
| 7351 | F | 73 | tAML | 2.18 | 44~46,XX,-7,-11,-17,+2mar[12]/46,XX[7] |
| 7479 | M | 73 | sAML | 1.32 | 86~91,XXY,-Y,-2,-3,-3,-4,-5,-6,-7,-8,-10,-10,-11,-12,-14,-16,-16,-17,-17,-18,-21,-22,+11~19mar[cp11]/46,XY[3] |
| 7484 | F | 78 | sAML | 17.45 | 50~51,XX,+1,del(5)(q15q33),+6,+8,+21[cp20] |
| 7680 | M | 72 | sAML | 4.14 | 43~46,XX,+1,del(3)(p14),-7,-13,add(16)(q22),17 [cp15] |
| 7754 | F | 74 | *de novo* | 11.50 | 43~45,X,-X,del(5)(q12q33),-12,-16,-17,+1~4mar [cp15] |
| 8074 | M | 77 | sAML | 4.27 | 45~46,XY,der(5)t(5;12)(5pter->5q14::12q13->12qter)[7],inv(5)(p15q13)[6],del(12)(q13)[7][cp7].ish der(5)t(5;12)(EGR1-,wcp12+)/46,XY[1] |
| 8189 | M | 68 | tAML | 7.33 | 34~45,X,-Y,-5,-7,-9,-12,-13,-14,-15,-16,-17,-18,-20,-22,+3~9mar[cp7]/46,XY[8] |
| 8281 | M | 75 | *de novo* | 4.11 | 44~47,XY,-2,del(3)(q21),del(5)(q12),-7,+?8,-10,-11,-13,-13,-15,-18,+3~6mar[cp13]/46,XY[2] |
| 8286 | F | 75 | sAML | 2.56 | 45,XX,-7[3]/46,XX[17] |
| 8353 | F | 59 | AML | 3.24 | 43~44,XX,del(5)(q31q35),-7,-18[cp19] |
| 8717 | M | 72 | *de novo* | 1.02 | 44~49,X,-Y,-4,-6,-7,-11,-17,-17,-18,+8~11mar[cp12]/46,XY[5] |
| 8851 | F | 78 | sAML | 2.60 | 45,XX,?del(5)(q13q32),del(6)(q25),-18,-20,-20,+2mar[cp6]/46,XX[3] |
| 8931 | M | 85 | tAML | 4.07 | 42~44,XY,t(1;2)(p34;p32)[4],der(5)t(5;13)(q11.2;q22)[16],dic(12;16)(p11.2;p11.2)[16],-13[16],-16[16]/46,XY[2] |
| 8239 | F | 46 | tAML | 16.15 | 41~44,XX,-7,-11,-12,-14,-16,-17,-21,-22,+5~6mar[cp20]/46,XX[1] |

**Supplementary Table 1:** **Clinical and cytogenetic data of 14 AML patients with somatic *TP53* mutations and one patient with Li-Fraumeni syndrome suffering from tAML (#8239).** Abbreviations: #, number; WBC, white blood cell count; F, female; M, male; *de novo*, *de novo* AML; tAML, therapy-related AML; sAML, secondary AML.

**
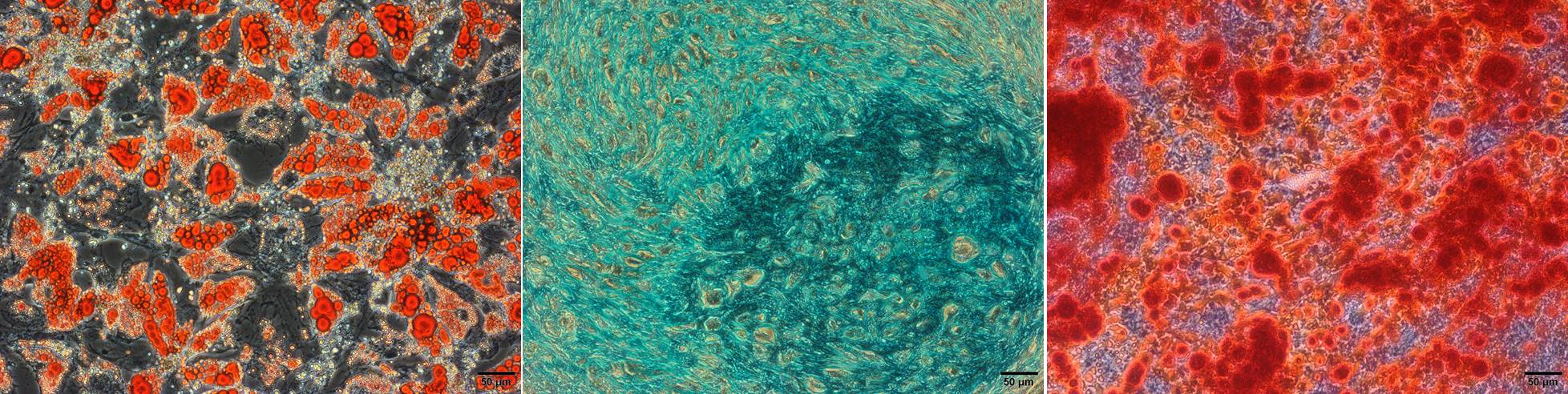
**

**Supplementary Figure 1. *In-vitro* differentiation of bone marrow-derived mesenchymal stromal cells of patients with *TP53* mutated AML.** Left, adipogenic differentiation (Oil Red O staining); middle, chondrogenic differentiation (Alcian Blue and Nuclear Fast Red staining); right, osteogenic differentiation (Alizarin Red S staining).

| **Sample #** | **Mutations** | **Cell type** | **VAF (%)** | **# mutated # wild-type**  **read groups** | |
| --- | --- | --- | --- | --- | --- |
| 7351 | *TP53*: c.614A>G, p.Y205C  NM_000546.3 | AML | 27.0 | 11.527 | 31.180 |
|  |  | BM-MSC | 0.0 | 0 | 14.428 |
| 7479 | *TP53*: c.646G>A, p.V216M | AML | 79.6 | 12.005 | 3.071 |
|  |  | BM-MSC | 0.0 | 0 | 888 |
|  | *TET2*: c.100C>T, p.L34F  NM_001127208 | AML | 50.1 | 1.064 | 1.061 |
|  |  | BM-MSC | 49.1 | 25.219 | 26.106 |
| 7484 | *TP53*: c.469G>T, p.V157F | **AML** | **82.8** | **57.398** | **11.916** |
|  |  | **BM-MSC** | **0.2**  **0.2** | **39**  **6** | **18.077**  **3.075** |
|  | *DNMT3A*: c.2657A>G,  p.Q886R  NM_022552 | AML | 38.2 | 2.861 | 4.634 |
|  |  | BM-MSC | 0.0 | 3 | 7.772 |
| 7680 | *TP53*: c.710T>A, p.M237K | AML | 89.8 | 15.725 | 1.778 |
|  |  | BM-MSC | 0.0 | 0 | 18.088 |
|  | *DNMT3A*: c.1979A>G,  p.Y660C | AML | 47.0 | 390 | 439 |
|  |  | BM-MSC | 0.0 | 0 | 27974 |
|  | *KRAS*: c.173C>T, p.T58I  NM_033360 | AML | 42.3 | 112.294 | 153.261 |
|  |  | BM-MSC | 0.0 | 0 | 2.148 |
|  | *CEBPA*: c.588_589GG>TT  NM_001285829 | AML | 46.0 | 28.486 | 33.399 |
|  |  | BM-MSC | 0.0 | 0 | 13.607 |
| 7754 | *TP53*: c.818G>A, p.R273H | AML | 75.3 | 952 | 313 |
|  |  | BM-MSC | 0.0 | 0 | 50.790 |
|  | *TP53*: c.749C>T, p.P250L | AML | 7.1 | 262 | 3.444 |
|  |  | BM-MSC | 0.0 | 1 | 6.589 |
|  | *NRAS*: c.35G>C, p.G12A  NM_002524.5 | AML | 1.8 | 6.353 | 346.729 |
|  |  | BM-MSC | 0.0 | 16 | 418.482 |
|  | *NRAS*: c.38G>A, p.G13D | AML | 1.5 | 5.256 | 346.140 |
|  |  | BM-MSC | 0.0 | 0 | 1.945 |
| 8074 | *TP53*: c.743G>A, p.R248Q | AML | 34.6 | 948 | 1.794 |
|  |  | BM-MSC | 0.0 | 3 | 18.070 |
| 8189 | *TP53*: c.395A>G , p.K132R | AML | 79.5 | 3.642 | 939 |
|  |  | BM-MSC | 0.0 | 0 | 2.343 |
|  | *NRAS*: c.183A>T, p.Q61H | AML | 24.5 | 407 | 1.255 |
|  |  | BM-MSC | 0.0 | 2 | 39.880 |
|  | *TET2*: p.S1848* | AML | 4.7 | 13.272 | 271.507 |
|  |  | BM-MSC | 0.0 | 1 | 20.812 |
| 8281 | *TP53*: c.824G>A, p.C275Y | AML | 35.6 | 96 | 174 |
|  |  | BM-MSC | 0.0 | 0 | 5.177 |
| 8286 | *TP53*: c.434T>C, p.L145P | AML | 12.5 | 202 | 1.413 |
|  |  | BM-MSC | 0.0 | 0 | 11.321 |
| 8353 | *TP53*: c.97-1G>A, p.S33fs | AML | 53.7 | 1.716 | 1.477 |
|  |  | BM-MSC | 0.0 | 0 | 3.186 |
|  | *RUNX1*: c.292delC, p.L98fs  NM_001754.4 | AML | 31.4 | 100 | 218 |
|  |  | BM-MSC | 0.0 | 0 | 16.155 |
| 8717 | *TP53*: c.838A>G, p.R280G | AML | 91.2 | 234.781 | 22.560 |
|  |  | BM-MSC | 0.0 | 0 | 55.922 |
| 8851 | *TP53*: c.746G>C, p.R249T | **AML** | **45.3** | **16.935** | **20.409** |
|  |  | **BM-MSC** | **0.2**  **0.1** | **453**  **28** | **241.361**  **28.400** |
|  | *TP53*: c.393_395del,  p.N131K | AML | 44.7 | 9.595 | 11.857 |
|  |  | BM-MSC | 0.0 | 0 | 2.407 |
| 8931 | *TP53*: c.841G>T, p.D281Y | AML | 68.9 | 1.949 | 880 |
|  |  | BM-MSC | 0.0 | 2 | 80.637 |
|  | *TET2*: c.1924C>T, p.Q642* | AML | 36.4 | 63 | 110 |
|  |  | BM-MSC | 0.0 | 0 | 868 |
| 8239 | *TP53*: c.467G>C, p.R156P | **AML** | **89.8** | **90.165** | **10.213** |
|  |  | **BM-MSC** | **47.1** | **91.058** | **102.077** |

**Supplementary Table 2. Analysis of *TP53* and cooperating mutations in AML specimens and cultured bone marrow-derived mesenchymal stromal cells (BM-MSCs)**. The *TET2* single nucleotide polymorphism c.100C>T, p.L34F (rs111948941), found in specimen #7479, was also detected in purified MSCs. Specimen #8239 served as a positive control and was derived from a patient with Li-Fraumeni syndrome suffering from therapy-related AML. Abbreviations: #, number; VAF, variant allele frequency.
